# Determinants of diabetes ketoacidosis among diabetes mellitus patients at North Wollo and Waghimra zone public hospitals, Amhara region, Northern Ethiopia

**DOI:** 10.1186/s12902-021-00692-y

**Published:** 2021-02-18

**Authors:** Addisu Getie, Adam Wondmieneh, Melaku Bimerew, Getnet Gedefaw, Asmamaw Demis

**Affiliations:** 1 Department of Nursing, College of Health Sciences, Woldia University, P.O.Box: 400, Woldia, Ethiopia; 2 Department of Midwifery, College of Health Sciences, Woldia University, P.O.Box: 400, Woldia, Ethiopia

**Keywords:** Diabetes ketoacidosis, Diabetes mellitus, North Wollo and Waghimra zone

## Abstract

**Background:**

Diabetes Mellitus (DM) is a metabolic disorder associated with acute and chronic complications. Diabetic ketoacidosis (DKA) is the most serious diabetic emergency in patients with type one and type two diabetes mellitus. It is the leading cause of mortality in children and young adults. Even though the burden of DKA has increased, no research has been conducted on the determinants of Diabetes ketoacidosis in Ethiopia, particularly in the Amhara region. Thus, this study aimed to identify the determinants of diabetes Ketoacidosis among Diabetes Mellitus patients at North Wollo and Waghimra Zone public Hospitals.

**Methods:**

An institution-based unmatched case-control study design was employed among 408 patients at North Wollo and Waghimra Zone Public Hospitals from March 1st to April 30th, 2020. A consecutive sampling method was used to select study participants. The data were collected using structured interviewer-administered questioners and reviewing of patient charts. The analysis was done using a binary logistic regression model. Then, *P*-value < 0.05 was considered statistically significant.

**Result:**

The mean (±SD) age of the study participants was 46.96 (± 15.175 SD) years. Irregular follow-up in diabetes clinic (AOR:4.19, 95% CI: 2.28–7.71), not received diabetic education (AOR: 2.87, 95% CI:1.44–5.72), alcohol drinking (AOR:2.99, 95% CI: 1.46–6.12), discontinuation of medications (AOR: 4.31, 95% CI:1.92–9.68), presence of comorbidity (AOR:2.57, 95% CI: 1.37–4.84), and being type one of diabetes mellitus (AOR: 2.01, 95% CI:1.11–3.63) were determinant factors of diabetic ketoacidosis .

**Conclusions:**

This study showed that the behavioral and clinical characteristics of diabetic patients were determinant factors of DKA. Follow-up in the diabetic clinic, diabetic education, discontinuation of medications, alcohol drinking, presence of comorbidities, and type of diabetes mellitus were independent determinants of diabetic ketoacidosis.

**Supplementary Information:**

The online version contains supplementary material available at 10.1186/s12902-021-00692-y.

## Introduction

Diabetes Mellitus is a group of common metabolic disorders characterized by the presence of chronic hyperglycemia, which is accompanied by greater impairment in the metabolism of carbohydrates, lipids, and proteins [1]. It is the leading cause of end-stage renal disease, traumatic lower extremity amputations, adult blindness, and predisposing to cardiovascular diseases [2]. Diabetic ketoacidosis (DKA) is one of the most common life-threatening acute metabolic complications of diabetes, which is usually associated with disability, reduced life expectancy, and causes enormous health costs for every society [3]. Diabetic ketoacidosis is thought to typify as type one diabetes mellitus. However, it has been reported to affect patients with type two diabetes mellitus. Although individuals with type one diabetes are more acidotic, type two diabetes patients also required treatment for acidosis as they can develop DKA [4]. A relative or absolute deficiency of insulin can cause increased counter regulatory hormones like cortisol, glucagon, growth hormone, and catecholamine, which enhances glycogenolysis and gluconeogenesis that results in hyperglycemia [5].. Thus, the presence of chronic hyperglycemia, accumulation of large quantities of ketone bodies, and subsequent metabolic acidosis can cause diabetic ketoacidosis [6]. Diabetic ketoacidosis reoccurs frequently due to poor adherence to insulin therapy [7]. Individuals who are hyperglycemic at the time of admission are at high risk of mortality, morbidity, and prolonged hospital stay [8]. Diabetic ketoacidosis is the leading cause of mortality in children and young adults, accounting for ~ 50% of deaths and half of all deaths are among patients younger than 24 years of age [6]. Treatment of DKA uses a large number of resources, accounting for an estimated total cost of $2.4billion annually [7].

In Africa, there is a dramatic increase in the prevalence of diabetic ketoacidosis in both rural and urban settings, and affecting both genders proportionally, which accounts for 3.2%. The inability of affording insulin treatment leads the patient to seek alternative treatment from traditional healers and using herbal medicine. The treatment guideline for DKA currently used may not be ideal as it is adapted from the developed world. These conditions can be complicating the disease condition and cause death in Africa. In the developing world, treatment due to infectious conditions can cause life-threatening drug interaction as the patients are on diabetic treatment [9].

In Ethiopia, DKA is increasing in the past two to three decades, becoming a major social and economic burden regarding bed occupancy, drug, and other resources needed for treatment of DKS. This is due to poor blood glucose monitoring, lack of follow-up, and use of a substance [3]. Limited resources and scarce inpatient facilities, presence of concomitant disease, and aging cause increased patient morbidity and mortality in Ethiopia [10]. Hypertension and diabetic neuropathy co-exist with diabetic ketoacidosis, which accounts for 78.9 and 43.7%, respectively [11].

Despite all challenges and costs incurred by diabetes and its complications, studies are limited to the overall determinants in Ethiopia. Therefore, identification of determinants of the disease and the possible preventive measures that could be instituted to arrest or delay the onset of diabetic ketoacidosis. Thus, this study was aimed to assess the possible determinants of diabetic ketoacidosis among diabetic patients in North Wollo and Waghimra Zone Public Hospitals.

## Methods

### Study design and setting

The unmatched case-control study design was conducted in North Wollo and Waghimra Zone Public Hospitals from March 1st to April 30, 2020. North Wollo zone founds in Amhara regional state with a capital city of Woldia that found 521 km away from Addis Ababa. There are six hospitals found in North Wollo and Waghimra Zone which are Woldia comprehensive specialized hospital, Kobo primary hospital, Lalibela general hospital, Mekiet primary hospital, Wadila primary hospital, and Tefera Hailu memorial hospital. The hospitals provide general and specialized treatment, known to be open 24 h for emergency services and each of them is assumed to be more than five million peoples.

### Population

All diabetes patients who were receiving treatment at public hospitals in North Wollo and Waghimra Zone were considered as a source population. Both type one and type two diabetes patients were included in the study. Diabetic ketoacidosis patients aged 18 and above were included as a case and those diabetic patients without diabetic ketoacidosis were considered as a control group. However, newly diabetic ketoacidosis diagnosed patients, critically and mentally ill patients who cannot give a response were excluded from the study in both case and control groups.

### Sample size determination

The sample size was determined using the double proportion formula with computer-based Epi info7 software Stat Cal by using the proportion difference approach. Among several exposure variables, selection of the appropriate exposure variables in controls was done based on the main interest variables of cases of the determinant for diabetic ketoacidosis using the assumptions; 95% confidence interval (Zα/_2_ = 1.96), power of 80% (Zβ =0.84), case to control ratio 1:3, the odds ratio to be detected ≥2. Then the calculated sample size was 371 [12]. Thus, adding 10% of the sample size to the larger sample size 371, the final required sample size was 408 (102 cases and 306 controls).

### Sampling procedure and sampling technique

All hospitals found in the North Wollo and Waghimra zone were included in the study. The total sample size was proportionally allocated to the number of diabetic patients with diabetic ketoacidosis for cases and without diabetic ketoacidosis for controls in each hospital. Then, the study participants from each hospital were selected by a consecutive sampling technique.

### Data collection tools and procedures

The data were collected by using structured interviewer-administered questionnaires and a review of case records of the hospital after the cases and controls were identified. In this study, the cases were diabetic patients who developed DKA. Diabetic ketoacidosis was diagnosed based on the case definition as blood glucose level > 250 mg/dl, serum ketone > 3 mmol/L or significant ketonuria more than + 2, and bicarbonate (HCO3^−^) < 15 mmol/L. The questionnaires were addressed the socio-demographic determinants, behavioral, and personal related determinants of diabetic ketoacidosis. A checklist consisting of clinical related determinants of diabetic ketoacidosis was also prepared to review the medical records of patients. The questioners were both open and close-ended and were prepared in English and translated to Amharic, which is a local language. Six BSc degree nurses were recruited as a data collector and three MSc nurses were supervisors who have experience in data collection.

### Data quality control

The questionnaires were pretested on 10% of the sample size at Dessie Referral Hospital. The data collectors and supervisors were trained before actual data collection, and the principal investigator and supervisors have supervised the data collectors closely. During data collection, both supervisors and data collectors checked the data for its completeness and missing information at each point. Furthermore, the data were also checked during and after entry into the computer before analysis.

### Data processing and analysis

The data were coded, cleaned, edited, and entered into Epi data version 4.2.0.0 to minimize logical errors and design skipping patterns. Then, the data were exported to SPSS version 24 for analysis. Summary statistics (mean or median) for continuous variables and percentage and frequency for categorical variables were computed for case and control groups separately. Tables and figures were used to present the data. The Bivariable analysis was used to see the association between each determinant factors and the outcome variable. The goodness of fit was tested by the Hosmer-Lemeshow statistic test. All variables with a *P*-value ≤0.25 in the Bivariable analysis were included in the final model of multivariable analysis to control all possible confounders. A multi co-linearity test was carried out to see the correlation between independent variables by using standard error (standard error > 2 was considered as suggestive of the existence of multi co-linearity). Adjusted odds ratios along with 95% CI were estimated and *P*-value < 0.05 was considered statistically significant.

### Ethical approval and consent form

Ethical clearance was obtained from Woldia University, College of Health Sciences, Research and Community Service Review Committee (RCSRC). A formal letter of permission and support was written to RHB from Woldia University and finally to selected health facilities. The study participants were informed about the purpose of the study, their right to refuse. Written and signed voluntary consent were obtained from study subjects before distributing the questionnaire. The respondents were also told that the information obtained from them was treated with complete confidentiality and did not cause any harm to them. All methods were carried out in accordance with relevant guidelines and regulations.

## Result

### Socio-demographic characteristics

A total of 408 (102 cases and 306 controls) diabetic patients were included in the study, of which 241 (59.1%) were males and 167 (40.9%) were females. The age range was from 18 to 80 years old with a mean and standard deviation of 46.96 ± 15.175 years, and the majority of respondents were found in the age range of 40–50 years (26.7%) (Table 1).

**Table 1:** Socio-demographic Characteristics of Diabetes mellitus patients admitted in North Wollo and Waghimra Zone public hospitals, 2020 (*n* = 408)

Variable		Frequency (N)	Percentage (%)
Age category	18–30	46	11.3
	31–40	95	23.3
	41–50	109	26.7
	51–60	82	20.1
	> 60	76	18.6
Marital status	Single	82	20.1
	Married	235	57.6
	Divorced	67	16.4
	Widowed	24	5.9
Religion	Orthodox	286	70.1
	Muslim	93	22.8
	Protestant	18	4.4
	Other	11	2.7
Residence	Urban	139	34.1
	Rural	269	65.9
Ethnicity	Amhara	274	67.2
	Tigray	78	19.1
	Oromo	44	10.8
	Other^a^	12	2.9
Level of education	Can’t read and write	123	30.1
	Read and write	128	31.4
	Primary school	72	17.6
	Secondary school	53	13.0
	College and above	32	7.8
Occupation	Housewife	117	28.7
	Government employee	37	9.1
	Farmer	124	30.4
	Student	48	11.8
	Merchant	42	10.3
	Privet workers	35	8.6
	Other	5	1.2

Other: Daily labor and drivers, other^a^: Guragie, Sidama, and Agew

### Behavioral and clinical characteristics

Of the 408 participants, 65.9% were living with diabetes mellitus longer than five years and 39% were overweight during diagnosis. Only 37.7% of the participants performed regular physical exercise and 51.2% had an infection. Regarding the type of diabetes mellitus (DM), 32.6% were type one (Table S1).

### The proportion of determinants of diabetic ketoacidosis among cases and controls

The onset of diabetes mellitus was highest among patients with the age range of 41–50 years old (31.4%). Of all cases, 82.4% had greater than five years of diabetic duration and more than 54% were overweight during diagnosis. Cases were more exposed almost for all determinant factors than controls. Behavioral determinant factors like alcohol drinking and cigarette smoking were higher among diabetic patients with diabetic ketoacidosis than those without diabetic ketoacidosis. More than 69% of cases did not perform regular physical exercise, 62.7% did not have a regular follow-up in the diabetic clinic, and 90.2% discontinued their medication. Only 17.6% of cases were received diabetic education. Similarly, infections were more common among cases (72.5%) than controls (44.1%). The presence of comorbidity was also higher among diabetes mellitus patients with diabetic ketoacidosis (67.6%) than patients without diabetic ketoacidosis (48%). There was also a difference between the types of diabetes mellitus among cases and control groups. Regarding the type of diabetes mellitus, 43% of diabetic ketoacidosis were diagnosed with type one diabetes mellitus (Table 2).

**Table 2:** Proportion of determinant factors among cases and controls among Diabetes mellitus patients admitted in North Wollo and Waghimra Zone public hospitals, 2020

Variable		Case		Control	
		N	%	N	%
Marital status	Single	8	7.8	74	24.2
	Married	69	67.6	166	54.2
	Divorced	15	14.7	52	17.0
	Widowed	10	9.8	14	4.6
Age at onset of DM	≤30	26	25.5	111	36.3
	31–40	26	25.5	74	24.2
	41–50	32	31.4	80	26.1
	51–60	8	7.8	23	7.5
	> 60	10	9.8	18	5.9
Duration of DM	1–5 year	18	17.6	121	39.5
	> 5 year	84	82.4	185	60.5
Body Mass Index	Underweight	10	9.8	54	17.6
	Normal	36	35.3	149	48.7
	Overweight	56	54.9	103	33.7
Regular follow up in DM clinic	Yes	38	37.3	225	73.5
	No	64	62.7	81	26.5
Received DM counseling’s	Yes	18	17.6	162	52.9
	No	84	82.4	144	47.1
Alcohol drinking	Yes	26	25.5	33	10.8
	No	76	74.5	273	89.2
Cigarette smoking	Yes	2	2.0	2	0.7
	No	100	98.0	304	99.3
Regular physical exercise	Yes	31	30.4	123	40.2
	No	71	69.6	183	59.8
Discontinue medication	Yes	92	90.2	136	44.4
	No	10	9.8	170	55.6
Emotional disturbance	Yes	76	74.5	179	58.5
	No	26	25.5	127	41.5
Presence of infection	Yes	74	72.5	135	44.1
	No	28	27.5	171	55.9
Type of treatment	OHA	18	17.6	59	19.3
	Insulin	84	82.4	247	80.7
Presence of comorbidity	Yes	69	67.6	147	48.0
	No	33	32.4	159	52.0
Type of DM	Type I	44	43.1	89	29.1
	Type II	58	56.9	217	70.9

*MD* Diabetes Mellitus, *OHA* Oral Hypoglycemic Agents, *Type I* Type one, Type II: Type two

### Admission blood glucose level

**Fig. 1:**
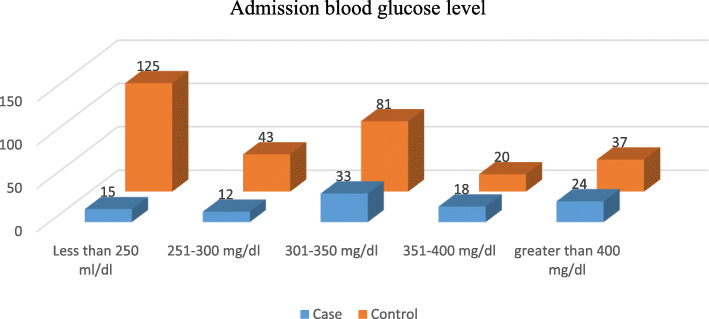
Admission blood glucose level of Diabetes mellitus patients admitted in North Wollo and Waghimra Zone public hospitals, 2020

Of all diabetes mellitus patients, 34.3% were less than 250 mg/dl of admission blood glucose level, and nearly 15% of patients had admission blood glucose levels greater than 400 mg/dl. Admission blood glucose levels less than 250 mg/dl were found among 14.7% of the cases, while 40.8% of controls had admission blood glucose levels less than 250 mg/dl. Conversely, 23.5% of cases had greater than 400 mg/dl admission blood glucose level, whereas only 12.1% of controls had a blood glucose level of greater than 400 mg/dl (Fig. 1).

### Baseline comorbidities

**Fig. 2:**
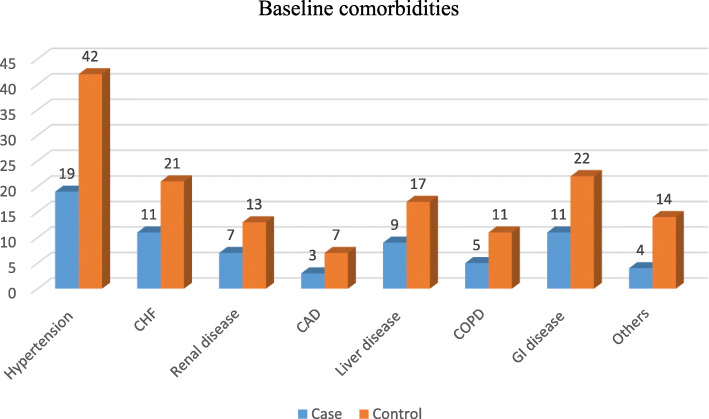
Baseline comorbidities of Diabetes mellitus patients admitted in North Wollo and Waghimra Zone public hospitals, 2020

Of all 408 diabetes mellitus patients, 52.9% had baseline comorbidities. Among those diabetes mellitus patients with baseline comorbidities, 67.6% were cases and 48% were controlled. Hypertension was found the highest comorbid, 28.2% of all diabetes mellitus patients with baseline comorbidities. Of these hypertensive patients, 27.5% were cases, whereas 28.6% were controls (Fig. 2).

### Determinant factors of diabetic ketoacidosis

This study identifies different behavioral and clinical factors as determinants of diabetic ketoacidosis. These determinant factors were the presence of regular follow-up in the diabetic clinic, received diabetic education, alcohol drinking, discontinuation of medications (insulin and oral hypoglycemic agents), presence of comorbidities, and type of diabetes mellitus. Diabetes mellitus patients who did not have a regular follow-up in the diabetic clinic were 4.19 times more likely to develop diabetic ketoacidosis than those who had regular follow-up [AOR: 4.19; 95% CI (2.28–7.71)]. Similarly, diabetes mellitus patients who did not receive diabetic education were 2.87 times more likely to develop diabetic ketoacidosis than patients who received diabetic education [AOR: 2.87; 95% CI (1.44–5.72)]. The odds of diabetic ketoacidosis among diabetes mellitus patients who drank alcohol was 2.99 times higher than diabetes mellitus patients who did not drink alcohol [AOR: 2.99; 95% CI (1.46–6.12)]. Regarding medication, patients who discontinued their medication were 4.31 times more likely to develop diabetic ketoacidosis than those who did not discontinue their medication [AOR: 4.31; 95% CI (1.92–9.68)]. The odds of diabetic ketoacidosis among diabetes mellitus patients who had comorbidities were 2.57 times higher than their counterparts [AOR: 2.57; 95% CI (1.37–4.84)]. Concerning the type of diabetes mellitus, patients with type one diabetes mellitus were 2.01 times more likely to develop diabetic ketoacidosis than patients with type two diabetes mellitus [AOR: 2.01; 95% CI (1.11–3.63)] (Table 3).

**Table 3:** Binary and Multiple Logistic Regression Analysis for determinants of diabetic ketoacidosis among Diabetes mellitus patients admitted in North Wollo and Waghimra Zone public hospitals, 2020

Variable		Case	Control	COR(95% CI)	AOR(95% CI)
Marital status	Single	8	74	1	1
	Married	69	166	3.85 (1.76–8.40)	1.89 (0.66–3.23)
	Divorced	15	52	2.67 (1.05–6.75)	0.87 (0.82–2.74)
	Widowed	10	14	2.88 (1.09–7.63)	1.87 (0.34–5.65)
Duration of DM	1–5 year	18	121	1	1
	> 5 year	84	185	3.05 (1.75–5.33)	2.42 (0.72–4.78)
Body Mass Index	Normal	36	149	1	1
	Underweight	10	54	0.77 (0.36–1.65)	1.73 (0.70–4.28)
	Overweight	56	103	2.25 (1.38–3.67)	1.89 (0.02–3.50)
Regular follow up in the diabetic clinic	No	64	81	4.68 (2.91–7.52)	**4.19 (2.28–7.71)**
	Yes	38	225	1	1
Received DM education	No	84	144	5.25 (3.01–9.16)	**2.87 (1.44–5.72)**
	Yes	18	162	1	1
Alcohol drinking	Yes	26	33	2.83 (1.56–5.02)	**2.99 (1.46–6.12)**
	No	76	273	1	1
Regular physical exercise	No	71	183	1.54 (0.95–2.49)	1.34 (0.82–2.07)
	Yes	31	123	1	1
Discontinue medication	Yes	92	170	7.36 (3.69–14.68)	**4.31 (1.92–9.68)**
	No	10	136	1	1
Emotional disturbance	Yes	76	179	2.07 (1.26–3.42)	1.36 (0.68–2.75)
	No	26	127	1	1
Presence of infection	Yes	74	135	3.35 (2.05–5.46)	1.99 (0.61–2.07)
	No	28	171	1	1
Presence of comorbidity	Yes	69	147	2.26 (1.13–4.33)	**2.57 (1.37–4.84)**
	No	33	159		1
Type of DM	Type I	44	89	1.85 (1.16–2.94)	**2.01 (1.11–3.63)**
	Type II	58	217	1	1

*AOR* Adjusted Odds Ratio, *CI* Confidence Interval, *COR* Crude Odds Ratio, *DM* Diabetes Mellitus, *Type I* Type one, *Type II* Type two

## Discussion

This institutional-based unmatched case-control study was conducted in North Wollo and Waghimra Zone public hospitals with diabetes mellitus patients to investigate the determinant factors of diabetic ketoacidosis. The literature identified different determinant factors of diabetic ketoacidosis [2, 13–15]. The results of this study identified determinants of diabetic ketoacidosis among diabetes mellitus patients. Follow-up in the diabetic clinic, receiving diabetic education, alcohol drinking, discontinuation of medication, presence of comorbidity, and type of diabetes mellitus were found determinant factors of diabetic ketoacidosis among diabetes mellitus patients. This study showed that the presence of follow-up in the diabetic clinic was found a determinant factor of diabetic ketoacidosis among diabetes patients. Diabetes mellitus patients who did not have a regular follow-up in the diabetic clinic were nearly four times more likely to develop diabetic ketoacidosis than patients who had a regular follow-up. This is in line with studies done in Southwest Ethiopia [16, 17]. The reason might be due to diabetes mellitus patients who had a regular follow-up in the diabetic clinic regularly knowing and monitors their blood glucose level which further prevents the development of diabetic ketoacidosis. Receiving diabetic education was also found a determinant factor of diabetic ketoacidosis. These findings were consistent with different studies done in selected hospitals of West Ethiopia [11, 18] and Northeast Ethiopia [19]. The reason might be due to patients who received education about diabetes mellitus can aware of the different blood glucose controlling mechanisms as they can easily prevent the occurrence of diabetic ketoacidosis. The finding of this study also revealed that alcohol drinking was a determinant factor of diabetic ketoacidosis. Diabetic Mellitus patients who drank alcohol were nearly three times more likely to develop diabetic ketoacidosis than those who did not drink alcohol. This is consistent with a study conducted in Atlanta [7]. Patients who discontinued their medication were 4.33 times more likely to develop diabetic ketoacidosis than patients who did not discontinue their medication. This finding agreed with studies conducted in Debre Markos referral hospital [15] and tertiary health care centers in Ethiopia [13]. This is because discontinuation of medications like oral hypoglycemic agents and insulin can increase the blood glucose level through decreasing tissue glucose uptake, increasing glucose absorption from the gastrointestinal tract, increasing gluconeogenesis and glycogenolysis, which results in lipolysis and cause diabetic ketoacidosis.

The presence of comorbidity was also a determinant factor of diabetic ketoacidosis which is supported by a study done in Hawassa comprehensive specialized hospital [12], Jima University Specialized Hospital [3], Shanan Gibe Hospital [11], and Jima Medical Center [14]. This might be because diabetes mellitus patients who have comorbidities, such as hypertension, chronic heart failure, gastrointestinal disturbance, renal disease, and others can directly or indirectly disturb the normal functions of insulin and other hormones which might be cause for the occurrence of diabetic ketoacidosis. The findings of this study also revealed that type one diabetes mellitus patients were more likely to develop diabetic ketoacidosis than those patients with type two diabetes mellitus. This is supported by different studies conducted at Tertiary hospitals in North east Ethiopia [2], Debre Markos referral hospital [15], West Ethiopia [18], and Jima Medical Center [14]. This is because patients with type one diabetes mellitus had a deficiency of insulin which causes the breakdown of lipid resulting in diabetic ketoacidosis, whereas type two diabetes mellitus patients had endogenous insulin which prevents lipolysis that can prevent the occurrence of diabetic ketoacidosis.

## Conclusion

This study identified different behavioral and clinical determinants of diabetic ketoacidosis among diabetes mellitus patients. Absence of regular follow-up in the diabetic clinic, not receiving diabetic education, discontinuation of medications, alcohol drinking, presence of comorbidities, and being a type one diabetes mellitus were independent determinants of diabetic ketoacidosis. Therefore, health professionals should intervene on the identified determinants of diabetic ketoacidosis to prevent the occurrence of diabetic ketoacidosis, know and manage the identified determinant factors of diabetic ketoacidosis, give health education regarding the determinant factors of diabetic ketoacidosis to all diabetic patients, and manage and prevent the possible baseline comorbidities. Furthermore, hospital administrators should emphasize and determine the determinant factors of diabetic ketoacidosis.

## Supplementary Information

**Additional file 1: Table S1**. Behavioral and clinical characteristics of Diabetes mellitus patients admitted in North Wollo and Waghimra Zone public hospitals, 2020.

## Data Availability

The datasets used for analysis are available from the corresponding author on reasonable request.

## Abbreviations

AOR: Adjusted Odds Ratio
BSc: Bachelor of Sciences
CI: Confidence Interval
COR: Crude Odds Ratio
DKA: Diabetic ketoacidosis
DM: Diabetes Mellitus
MSc: Master of Science
RCSRC: Research and Community Service Review Committee
SPSS: Statistical Package for Social Science
SD: Standard Deviation
