# Supplementary material for: Determinants of diabetes ketoacidosis among diabetes mellitus patients at North Wollo and Waghimra zone public hospitals, Amhara region, Northern Ethiopia
Source: BMC Endocr Disord. 2021 Feb 18;21:26. doi: 10.1186/s12902-021-00692-y (PMC7890609; doi:10.1186/s12902-021-00692-y)
Supplement: Supplementary file 1 — Additional file 1: Table S1. Behavioral and clinical characteristics of Diabetes mellitus patients admitted in North Wollo and Waghimra Zone public hospitals, 2020. [file 12902_2021_692_MOESM1_ESM.docx]

Table 1: Behavioral and clinical characteristics of Diabetes mellitus patients admitted in North Wollo and Waghimra Zone public hospitals, 2020 (n=408)

| Variable | | Frequency (n) | Percentage (%) |
| --- | --- | --- | --- |
| Age at onset of DM | ≤30 | 117 | 28.7 |
|  | 31-40 | 100 | 24.5 |
|  | 41-50 | 112 | 27.5 |
|  | 51-60 | 31 | 7.6 |
|  | >60 | 28 | 6.9 |
| Duration of DM | 1-5 year | 139 | 34.1 |
|  | >5 year | 269 | 65.9 |
| Body Mass Index | Underweight | 64 | 15.7 |
|  | Normal | 185 | 45.3 |
|  | Overweight | 159 | 39.0 |
| Regular follow up in DM clinic | Yes | 263 | 64.5 |
|  | No | 145 | 35.5 |
| Received DM counseling’s | Yes | 180 | 44.1 |
|  | No | 228 | 55.9 |
| Alcohol drinking | Yes | 59 | 14.5 |
|  | No | 349 | 85.5 |
| Cigarette smoking | Yes | 4 | 1.0 |
|  | No | 304 | 74.5 |
| Regular physical exercise | Yes | 154 | 37.7 |
|  | No | 254 | 62.3 |
| Discontinue medication | Yes | 228 | 55.9 |
|  | No | 180 | 44.1 |
| Emotional disturbance | Yes | 255 | 62.5 |
|  | No | 153 | 37.5 |
| Presence of infection | Yes | 209 | 51.2 |
|  | No | 199 | 48.8 |
| Type of treatment | OHA | 77 | 18.9 |
|  | Insulin | 331 | 81.1 |
| Type of DM | Type I | 133 | 32.6 |
|  | Type II | 275 | 67.4 |

OHA: Oral Hypoglycemic Agents, DM: Diabetes Mellitus, Type I: Type one, Type II: Type two.
